# Structure–Function Analysis of *Mycobacterium tuberculosis* Drug Target Cytochrome P450 125 (CYP125) Enzyme Family

**DOI:** 10.3390/ijms26178531

**Published:** 2025-09-02

**Authors:** Nompilo Masinga, David R. Nelson, Khajamohiddin Syed

**Affiliations:** 1 Department of Biochemistry and Microbiology, Faculty of Science, Agriculture and Engineering, University of Zululand, Empangeni 3886, South Africa; nompilosisekelo@gmail.com; 2 Department of Microbiology, Immunology and Biochemistry, University of Tennessee Health Science Center, Memphis, TN 38163, USA; drnelson1@gmail.com

**Keywords:** cytochrome P450, CYP125, cholesterol, active site, active site cavity shape, substrate specificity, crystal structures

## Abstract

Tuberculosis, the deadliest human lung disease caused by *Mycobacterium tuberculosis*, continues to be a global health threat, and finding new drugs and drug targets seems an ongoing battle. The cytochrome P450 CYP125A1 enzyme of *M. tuberculosis* H37Rv, which is involved in cholesterol metabolism, is a well-established target for drug development. Research is ongoing to identify new compounds that target this enzyme. Understanding the structure–activity relationship of CYP125 family members is crucial for developing a specific and efficient inhibitor. In this direction, this study analyzed 21 crystal structures of CYP125 family enzymes, unraveling the factors responsible for substrate specificity and the amino acids that play a key role in catalysis. One of the unique features of CYP125A1 is its active site cavity shape, which determines the specificity of substrates and inhibitors. The active site cavity is shaped like a letter box, lined by hydrophobic residues, and it transitions into a funnel-like shape with a progressive narrowing as it approaches the heme. Due to this shape, the cholesterol and cholest-4-en-3-one serve as substrates, but not androstenedione, as the former molecules have an alkyl side chain that extends down the narrow funnel channels, interacting with the heme iron. Different binding patterns were observed for substrates and indole-derived inhibitors. Both type I and type II interactions were observed with the non-azole P450 inhibitor LP10 and indole-derived compounds, where the side chain of the indole-derived compound determined the type of interaction. This study provides a comprehensive understanding of the structure–function analysis of P450 enzymes and the interactions of CYP125A members with various ligands. Our findings pave the way for designing new and specific CYP125A1 inhibitors that will ultimately be developed into novel anti-TB drugs.

## 1. Introduction

Tuberculosis (TB) is the deadliest human disease caused by *Mycobacterium tuberculosis*, mainly targeting the human lungs and potentially progressing into a chronic, systemic wasting condition, which continues to be a major global public health concern, contributing to significant illness and death worldwide [1,2]. In 2023, a total of 8.2 million people were newly diagnosed with TB worldwide, an increase from 7.5 million in 2022 [2]. South-East Asia and Africa were responsible for the highest percentage of TB cases worldwide in 2023 [2]. Africa was found to have the highest rate of HIV/TB co-infection, or those with both HIV and active TB, with South Africa accounting for an incredible 50% of these instances [3].

Despite the availability of treatment, TB control continues to face significant challenges due to the declining effectiveness of key first-line drugs isoniazid, streptomycin, rifampicin, ethambutol, and pyrazinamide, caused by the emergence of drug-resistant (isoniazid-resistant) and multidrug-resistant (MDR-resistant to rifampicin and isoniazid) and extensively drug-resistant (XDR-resistant to rifampicin plus any fluoroquinolone plus at least one of either bedaquiline or linezolid) *M. tuberculosis* strains [2]. This growing threat of drug resistance has become a serious global health concern. This problem has been further compounded by a long-standing innovation gap in the discovery of new anti-TB drugs [4]. To curb the spread of *M. tuberculosis* and reduce the increasing mortality rates associated with TB, identifying novel therapeutic targets has become a pressing priority.

Cytochrome P450 monooxygenases (CYPs/P450s) are a superfamily of heme-containing enzymes that catalyze different types of reactions with stereo- and regio-specificity [5,6,7,8]. P450s are found across the domains of life (Archaea, Bacteria, and Eukarya) and viruses [9,10], indicating their important role in organisms’ physiology. Genome-wide analysis of P450s in 2666 mycobacterial species revealed the presence of P450s in their genomes with *Mycolicibacterium rhodesiae* JS60 having the highest (95 P450s), and *Mycobacterium leprae* Br492 the lowest number of P450s (3 P450s) in their genomes [11]. P450s in mycobacterial species were found to play a role in cholesterol metabolism [12,13]. Genome sequencing of *M. tuberculosis* H37Rv revealed the presence of 20 P450s in its genome [14]. Research revealed that P450 enzymes such as CYP121A1, CYP125A1, and CYP142A1 are promising drug targets, and developing inhibitors against these P450s is in progress [15].

Among the drug targets, the CYP125A1 gene is part of an intracellular growth (*igr*) locus, which was found to play an essential role in the survival of *M. tuberculosis* [16,17]. Knockout of this locus resulted in the accumulation of cholest-4-en-3-one, preventing the growth of *M. tuberculosis* in a cholesterol-rich medium, and also in a medium where glucose, acetate, and glycerol served as a sole carbon source [16,17,18]. High-density mutagenesis and deep sequencing study identified that CYP125A1 is essential for the survival and growth of this pathogen on cholesterol [19]. Subsequent studies revealed that CYP125 P450s from different bacterial species, including from *M. tuberculosis* H37Rv, were indeed involved in not only the oxidation of cholesterol but also cholest-4-en-3-one, the first product of cholesterol oxidation by 3β-hydroxysteroid dehydrogenase [20,21,22] (Table 1). It has been found that CYP125s oxidize cholesterol and cholest-4-en-3-one at the 26- or 27-carbon, successively converting them into their corresponding alcohol, aldehyde, and then carboxylic acid products [23,24] (Table 1).

The activity analysis of CYP125s from different bacterial species revealed that almost all showed activity toward primary substrates such as cholesterol and cholest-4-en-3-one (Table 1). However, some substrate specificity was observed towards derivatives of cholesterol and cholest-4-en-3-one, indicating different roles played by these CYP125s in specific organisms (Table 1). Furthermore, CYP125s were also found to oxidize the flavonoid chrysin and the plant sterol sitosterol (Table 1), indicating that CYP125s may have a broader substrate activity. Due to the potential role of CYP125 as a drug target against *M. tuberculosis*, many studies are focused on identifying inhibitors against this P450 to develop them as anti-TB drugs.

A comprehensive structure–activity relationship analysis is crucial for developing inhibitors or enhancing the catalytic activities of enzymes, including P450s. In a recent study, a thorough analysis of 53 crystal structures of CYP121A1 revealed active site cavity dynamics and the role of amino acids in catalysis, which provided crucial information that can be used for designing new and specific CYP121A1 inhibitors that can be developed as anti-TB drugs [31]. Furthermore, an analysis of 44 CYP107 crystal structures revealed critical new information about this P450 family that can be used to engineer CYP107s to produce novel molecules of genetically biotechnological interest [32].

As CYP125A1 of *M. tuberculosis* is a drug target, research is underway to develop inhibitors against this P450 to develop novel anti-TB drugs [33,34]. Understanding the structure–activity of CYP125s provides novel insights similar to those we found for CYP121A1 of *M. tuberculosis* [31], that can be useful in developing novel and specific CYP125 inhibitors. However, a comprehensive analysis of the available crystal structures of CYP125s has not been reported. In this study, we address this research knowledge gap. We analyzed the crystal structures of 21 CYP125A members and delineated the structure–function relationships of CYP125, including a comprehensive analysis of its interactions with ligands (substrates and inhibitors).

## 2. Results and Discussion

### 2.1. CYP125A Members’ Active Site Amino Acids Are Highly Dynamic

Twenty-one CYP125A members’ crystal structures were retrieved from the Research Collaboratory for Structural Bioinformatics (RCSB) Protein Data Bank (PDB) [35] (Table 2). Analysis of CYP125A members’ crystal structures revealed that 71% of the resolved crystal structures are in the closed conformation (bound with ligand and heme), and the rest belong to the open conformation (containing only the heme cofactor) (Table 2). The most closed conformation crystal structures are expected, as CYP125 is a drug target, and one would like to see the ligand (either substrate or inhibitor) interactions to develop a potential drug against CYP125A.

The surface area of the active site cavity for open conformation CYP125A members’ crystal structures ranged between 972 Å^2^ and 1267 Å^2^, whereas the surface area of the active site cavity for closed conformation ranged between 892 Å^2^ and 1499 Å^2^ (Table 2). The volume of the active site cavity in an open conformation ranged between 560 Å^3^ and 786 Å^3^ compared to 487 Å^3^ and 859 Å^3^ in a closed conformation (Table 2). The average surface area and volume for CYP125As are 1135 Å^2^ and 668 Å^3^ in an open conformation, compared to 1067 Å^2^ and 586 Å^3^ in a closed conformation (Table 2). Therefore, the estimated change in surface area is 69 Å^2,^ and the volume is 82 Å^3^, reflecting that binding the ligands in the active site cavity decreases both the surface area and the volume, indicating a dynamic change in the active site cavity when a ligand is bound. A decrease in the surface area and volume is also observed for CYP107FH5 from open to closed conformation, and a decrease in surface area for CYP102A1 (Table 3). Compared to other P450s, the change in surface area of CYP125A is higher than that of CYP121A1, and the volume change is higher than that of CYP121A1 and CYP102A1 (Table 3). In contrast to CYP125A, an increase in volume has been observed in human drug-metabolizing P450s, such as CYP3A4 and CYP2A6 [36,37]. CYP2A6 has a change of 251 Å^3^ to 300 Å^3^ when bound to phenacetin [36]; CYP3A4 has a change of 1173 Å^3^ to 2017 Å^3^ in volume from open to closed conformation when bound to ketoconazole, and 2682 Å^3^ when bound to erythromycin [37]. Structural dynamic analysis of amino acids in 19 CYP125A1 crystal structures revealed a root-mean-square difference (RMSD) of 0.6, and inclusion of CYP125A3 and CYP125A4 gave an RMSD of 0.7 (Table 3). The RMSD of CYP125A members are the lowest compared to CYP102A1 (RMSD of 4.4 Å), CYP107FH5 (RMSD of 3.0 Å), CYP109E1 (RMSD of 2.9 Å), and only slightly higher compared to CYP121A1 (RMSD of 0.2 Å), indicating CYP125A1 is somewhat flexible compared to CYP121A1 but not as much as CYP102A1, CYP109E1, and CYP107FH5 (Table 3).

**Table 2:** CYP125A P450s used in the study and their active site cavity characteristics. Ligand abbreviations are the same as listed at The Research Collaboratory for Structural Bioinformatics (RCSB) Protein Data Bank (PDB) [35].

P450 Name	Species Name	PDB Code	Ligand(s)	Resolution (Å)	Surface Area (Å^2^)	Volume (Å^3^)	Conformation	Reference(s)
CYP125A1	*Mycobacterium tuberculosis H37Rv*	2X5L	HEM	1.48	1117.598	680.452	Open	[38]
CYP125A1	*M. tuberculosis H37Rv*	2XN8	HEM	1.64	972.112	588.815	Open	[38]
CYP125A1	*M. tuberculosis H37Rv*	3IVY	HEM	1.35	1155.315	559.857	Open	[20]
CYP125A1	*M. tuberculosis H37Rv*	3IW0	HEM	1.70	1148.149	632.7	Open	[20]
CYP125A1	*M. tuberculosis H37Rv*	2XC3	RT8	1.50	1098.993	616.083	Closed	[38]
CYP125A1	*M. tuberculosis H37Rv*	7QKE	E1V	2.30	938.417	487.117	Closed	[34]
CYP125A1	*M. tuberculosis H37Rv*	7QNN	E93	2.47	954.942	542.408	Closed	[34]
CYP125A1	*M. tuberculosis H37Rv*	7QWN	DQE	1.93	1034.257	676.798	Closed	[34]
CYP125A1	*M. tuberculosis H37Rv*	7R1I	HIH	2.24	939.011	506.177	Closed	[34]
CYP125A1	*M. tuberculosis H37Rv*	7R3U	2QC	1.86	1245.124	604.994	Closed	[34]
CYP125A1	*M. tuberculosis H37Rv*	7YXF	I6Y	1.85	1136.627	696.791	Closed	[34]
CYP125A1	*M. tuberculosis H37Rv*	7ZQR	JK9	1.79	1498.941	859.045	Closed	[34]
CYP125A1	*M. tuberculosis H37Rv*	7ZLZ	JFC	1.89	994.537	517.368	Closed	[34]
CYP125A1	*M. tuberculosis H37Rv*	7ZSU	JV3	2.20	910.116	504.892	Closed	[34]
CYP125A1	*M. tuberculosis H37Rv*	7ZT0	JUR	1.99	1171.918	691.505	Closed	[34]
CYP125A1	*M. tuberculosis H37Rv*	7ZXD	KB9	2.09	1013.23	549.261	Closed	[34]
CYP125A1	*M. tuberculosis H37Rv*	2X5W	K2B	1.58	892.144	507.427	Closed	[24]
CYP125A1	*M. tuberculosis H37Rv*	3IW1	ASD	2.00	1035.662	491.798	Closed	[20]
CYP125A1	*M. tuberculosis H37Rv*	3IW2	EKO	2.19	1064.239	540.218	Closed	[20]
CYP125A4	*M. smegmatis MC2 155*	5DQN	HEM	2.26	1152.161	785.506	Open	[25]
CYP125A3	*M. smegmatis MC2 155*	4APY	HEM	2.00	1267.39	763.058	Open	[26]

Abbreviations: HEM: Protoporphyrin IX containing Fe, ASD: 4-androstene-3-17-dione, EKO: 4-[(2R)-2-[(4-chlorobenzyl) oxy]-2-(2,4-dichlorophenyl) ethyl]-1H-imidazole, I6Y: 1-(2-piperazin-1-ylethyl)-5-pyridin-4-yl-indole, JFC: ethyl 1-(2-morpholin-4-ylethyl)-5-pyridin-4-yl-indole-2-carboxylate, DQE: ethyl 5-pyridin-4-yl-1~(H)-indole-2-carboxylate, HIH: ethyl 1-(2-piperidin-4-ylethyl)-5-pyridin-4-yl-indole-2-carboxylate, 2QC: 1-[4-(1,2,3-thiadiazol-4-yl) phenyl] methanamine, JUR: 1-(2-piperazin-1-ylethyl)-5-pyridin-4-yl-indole-2-carboxamide, KB9: 1-[1-(2-piperidin-4-ylethyl)-5-pyridin-4-yl-indol-2-yl] butan-1-one, JK9: 4-(4-methoxyphenyl) pyridine, E1V: ethyl 1-(cyclohexylmethyl)-5-pyridin-4-yl-indole-2-carboxylate, E93: ethyl 1-(cyclopentylmethyl)-5-pyridin-4-yl-indole-2-carboxylate, JV3: methyl 3-pyridin-4-ylbenzoate, RT8: nalpha-[(trans-4-methylcyclohexyl)carbonyl]-n-pyridin-4-yl-d-tryptophanamide, K2B: (8alpha,9beta)-cholest-4-en-3-one.

**Table 3:** Comparative analysis of the surface area, volume, and root mean square deviation (RMSD) of four P450s.

P450 Name	Change in Average Surface Area (Å^2^) *	Change in Average Volume (Å^3^) *	RMSD (Å)	Reference
CYP107FH5	276 ^#^	494 ^#^	3.0	[32]
CYP121A1	37	8	0.2	[31]
CYP102A1	179 ^#^	23 ^#^	4.4	[39]
CYP109E1	151 ^#^	545	2.9	[40]
CYP125A	68 ^#^	82 ^#^	0.6 and 0.7 *	Current work

Symbols: ^#^, the active site surface area, and volume decreased from open to closed conformation. * Two RMSD values for CYP125A are indicated with (0.7) and without (0.6) inclusion of CYP125A3 and CYP125A4. The same parameter settings were used to generate the data for all P450s in the table, enabling accurate comparison.

**Figure 1:**
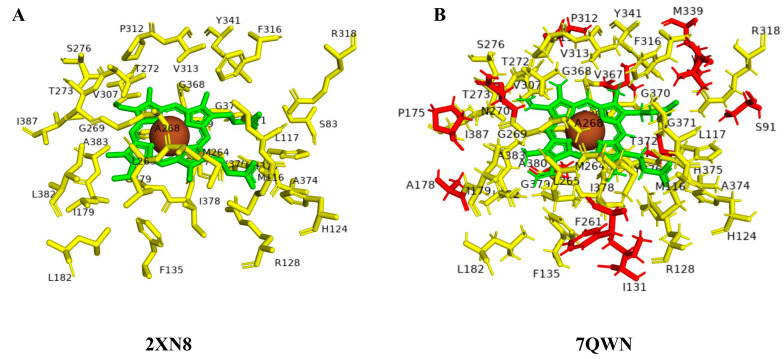
Active site cavity amino acid dynamics analysis between open (**A**) and closed (**B**) conformations of CYP125 P450s. Heme is shown in green, iron is shown as a brown sphere, amino acids that are common between open and closed conformations are shown in yellow, and amino acid residues unique to closed conformation are shown in red. Details on amino acids are presented in Table 4.

CYP125A members’ active site cavity analysis revealed the presence of 34 amino acids in all open conformations and 34 to 45 amino acids in closed conformations (Figure 1 and Table 4). The 34 amino acids in all CYP125 crystal structures in the open conformation are conserved, and 31 amino acids are conserved across the closed conformations, indicating their key role in maintaining the structure of the active site and possibly playing a role in catalysis (Figure 1 and Table 4). Comparison of closed conformation CYP125A crystal structures revealed the presence of unique amino acids in their active site cavities, ranging from 3 to 14 (Table 4), indicating the role of these amino acids in ligand binding. A comparative analysis between open and closed conformations revealed that the 34 amino acids found in the open conformation are also present in the closed conformation, further indicating that these amino acids play a role in maintaining the structure of the active site and in catalysis (Figure 1). Interestingly, 12 amino acids were uniquely found in a closed conformation, suggesting their role in interacting with different ligands or in catalysis (Figure 1 and Table 4).

### 2.2. The Shape of the Active Site Cavity Determines the Substrate Specificity

Crystal structures of CYP125A1 from *M. tuberculosis* with cholest-4-en-3-one, androstenedione, and the anti-tubercular compound econazole, as well as a CYP125A1 model with cholesterol, revealed details of its molecular mechanism of substrate specificity [20,24]. An open conformation of the CYP125A1 crystal structure revealed that its active site cavity is shaped like a letterbox, lined by hydrophobic residues, and it transitions into a funnel-like shape with a progressive narrowing as it approaches the heme [20]. The letterbox shape is ideally suited for binding a polycyclic sterol. The Val267 was found to play a role in determining the hexa-coordinate low-spin and penta-coordinate high-spin states of CYP125A1. The reorientation of this residue affected the heme distal pocket’s hydrogen bonding network and, consequently, the extent of water ligation with the heme. The heme penta-coordinate high-spin state, specifically the Val267 carbonyl backbone oxygen, formed an indirect hydrogen bond with the water molecule closest to the heme iron. Hence, substrate binding is predicted to reorient Val267 by displacing water molecules, thereby transforming CYP125A1 from a low-spin to a high-spin state.

**Figure 2:**
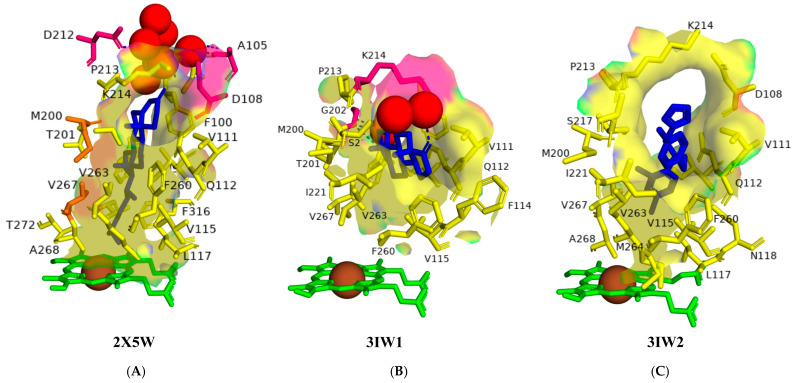
Analysis of CYP125A1 interactions with cholest-4-en-3-one (**A**), androstenedione (**B**), and econazole (**C**). Heme is shown in green, iron is shown as a brown sphere, ligand is shown in blue, amino acids that are part of the active site cavity are shown in yellow, and amino acid residues sharing van der Waals interactions with substrate are shown in orange. Amino acid residues sharing a direct and water-mediated polar interaction with the ligand are shown in pink. Polar interactions are indicated as blue dashed lines, water molecules are represented as red spheres, and amino acid residues are labelled according to their single-letter codes. The shape of the active site cavity is shown with a transparent solid surface. The PDB code is displayed under the structure. Amino acids found within 5 Å of the ligand are shown in Table 5.

Analysis of CYP125A1 interactions with cholesterol and cholest-4-en-3-one revealed the amino acids that play a role in the catalysis (Figure 2). CYP125A1 bound with cholest-4-en-3-one revealed that the sterol D-ring is positioned near the top of the active site cavity (Figure 2A). The hydrophobic substrate binding tunnel narrows as it approaches the catalytic site and is entirely encapsulated by the alkyl side chain of cholest-4-en-3-one. CYP125A1 model with cholesterol also suggests the same binding pattern, where the alkyl side chain is in a narrow funnel shape and close to the heme. Met200 and Val267, which protrude between the van der Waals space of the axially oriented C-18 and C-19 methyl groups, prevent cholest-4-en-3-one from sliding along the tunnel, potentially mitigating the electron-rich nature of the tertiary C-H bond and making it a more reactive target for selective oxidation (Figure 2). Interestingly, the terminal portion of the cholesterol side chain is in close contact with Val267. This interaction may be essential for promoting the conformational readjustment of the side chain to displace the distal water and trigger catalysis, as mentioned above. Trp414 practically locks cholest-4-en-3-one into the observed position on the other side of the tunnel by fitting into the corner between the aliphatic chain and the sterol D-ring [24].

The cholest-4-en-3-one forms a direct hydrogen bond with one water molecule, leading to water-mediated polar interactions with three additional water molecules (Figure 2A). The keto group does not form polar contacts with the amino acid residues but participates in an H-bonding network via two water molecules involving Ala105, Asp108, and Asp212 (Figure 2A). Val267 is close to the alkyl side chain, suggesting that a low-to-high spin transition from substrate binding causes the reorientation of Val267, as described above. These results indicate that cholest-4-en-3-one and cholesterol extend down the narrow binding funnel with the alkyl side chain terminal carbons reaching heme and thus facilitating their hydroxylation at C26.

Analysis of CYP125A1 interactions with androstenedione and econazole further revealed that the shape of the active site cavity determines the substrate specificity (Figure 2B,C). The binding of these molecules takes place within the active site cavity, but none of them are close to the heme iron because the active site’s funnel shape does not allow entry of these molecules close to the heme (Figure 2B,C) [20]. The C3-oxygen atom of the androstenedione forms polar contact with a water molecule, leading to water-mediated polar interactions with a water molecule and Lys214 (Figure 2B). The C17-oxygen atom also forms polar contact and water-mediated polar interaction with Gly202 and one water molecule (Figure 2B). Analysis of CYP125A1 interactions with econazole revealed binding patterns similar to those of androstenedione, both of which are confined to the letterbox cavity and cannot pass through the funnel-shaped cavity to reach the heme (Figure 2C). Because androstenedione and econazole do not have the alkyl side chain as cholesterol or cholest-4-en-3-one does, their binding pattern is incompatible with P450 oxidation. Additionally, the active site’s funnel-like shape prevents these two molecules from reaching the heme iron [20].

**Table 5:** List of amino acids surrounding the ligand within 5 Å in CYP125A P450s.

PDB Code	Amino Acids
2X5W	Ile97, Phe100, Ile104, **Asp108**, Val111, Gln112, Val115, Leu117, *Met200*, Thr201, Pro213, Lys214, Ser217, Phe260, Ala263, Met264, *Val267*, Ala268, Thr272, Val313, Phe316, Trp414
2XC3	Ile97, Phe100, Ile104, **Asp108**, Ile109, Val111, **Gln112**, Phe114, Val115, Leu117, **Asn118**, **Met200**, Thr201, Gly202, Pro213, Lys214, **Ser217**, Ile221, Phe260, Val263, Met264, Val267, Ala268, Thr272, Phe316, Trp414
3IW1	Ile97, Phe100, Val111, Gln112, Phe114, Val115, Met200, Thr201, **Gly202**, Pro213, **Lys214**, Ser217, Ile221, Phe260, Val263, Val267, Trp414
3IW2	Ile97, Asp108, Val111, Gln112, Val115, Leu117, Asn118, Met200, Pro213, Lys214, Ser217, Ala218, Ile221, Phe260, Val263, Met264, Val267, Ala268
7QKE	Ile97, Phe100, Ile104, Asp108, Ile109, Val111, Gln112, Phe114, Val115, Leu117, Asn118, Met200, Thr201, Asn203, Pro213, Lys214, Ser217, Ile221, Phe260, Val263, Met264, Val267, Ala268, Val313, Phe316, Trp414
7QNN	Ile97, Phe100, Ile104, Asp108, Val111, Gln112, Phe114, Val115, Leu117, Met200, Thr201, Pro213, Ser217, Ile221, Phe260, Val263, Met264, Val267, **Ala268**, Phe316, Trp414
7QWN	Ile97, Phe100, Gln112, Leu117, **Met200**, Thr201, Asn203, Val267, Ala268, Gly269, Asn270, **Glu271**, Thr272, Val313, Phe316, Cys377, Trp414, Leu415
7R1I	Ile97, **Arg99**, Phe100, Val111, Gln112, Val115, Leu117, Met200, Thr201, **Asn203**, Pro213, Ser217, Phe260, Val263, Met264, Val267, **Ala268**, Thr272, Val313, Phe316, **Trp414**
7R3U	Ile97, Gln112, Leu117, Met200, Thr201, Met264, Val267, Ala268, Gly269, Thr272, Val313, Phe316, Cys377, Trp414, Leu415
7YXF	Ile97, Phe100, Ile104, Asp108, Val111, Asn112, Leu117, Met200, Thr201, Gly202, Pro213, Lys214, Ser217, Met264, Val267, Ala268, Gly269, Asn270, Glu271, Thr272, Val313, Phe316, Cys377, Trp414, Leu415
7ZLZ	Ile97, Phe100, Ile104, Asp108, Val111, Gln112, Phe114, Val115, Leu117, Met200, Thr201, Gly202, Asn203, Pro213, Lys214, Ser217, Ile221, Phe260, Val263, Met264, Val267, **Ala268**, Thr272, Val313, Phe316, Trp414, Leu415
7ZQR	Ile97, Leu117, Met200, Thr201, Val267, Ala268, Thr272, Val313, Phe316, Cys377, Trp414, Leu415
7ZSU	Ile97, Gln112, Leu115, Val115, Leu117, Asn118, Met200, Thr201, Met264, Val267, Ala268, Gly269, Glu271, Thr272, Val313, Phe316, Cys377, Trp414
7ZT0	Ile97, Phe100, Gln112, Leu117, Met200, Thr201, Gly202, Asn203, Pro213, Val267, Ala268, **Glu271**, Thr272, Val313, Phe316, Cys377, Trp414, Leu415
7ZXD	Ile97, **Arg99**, Phe100, Val111, Gln112, Val115, Leu117, Met200, Thr201, **Asn203**, **Glu204**, Pro213, Ser217, Ile221, Phe260, Val263, Met264, Val267, **Ala268**, Thr272, Val313, Phe316, Trp414

Note: Amino acids in bold represent direct polar/water-mediated interactions, amino acids in italics represent van der Waals interactions, and amino acids underlined represent hydrophobic interactions.

### 2.3. CYP125A1 Interactions with the LP10 Molecule Revealed Structural Determinants for Developing Selective Inhibitors

**Figure 3:**
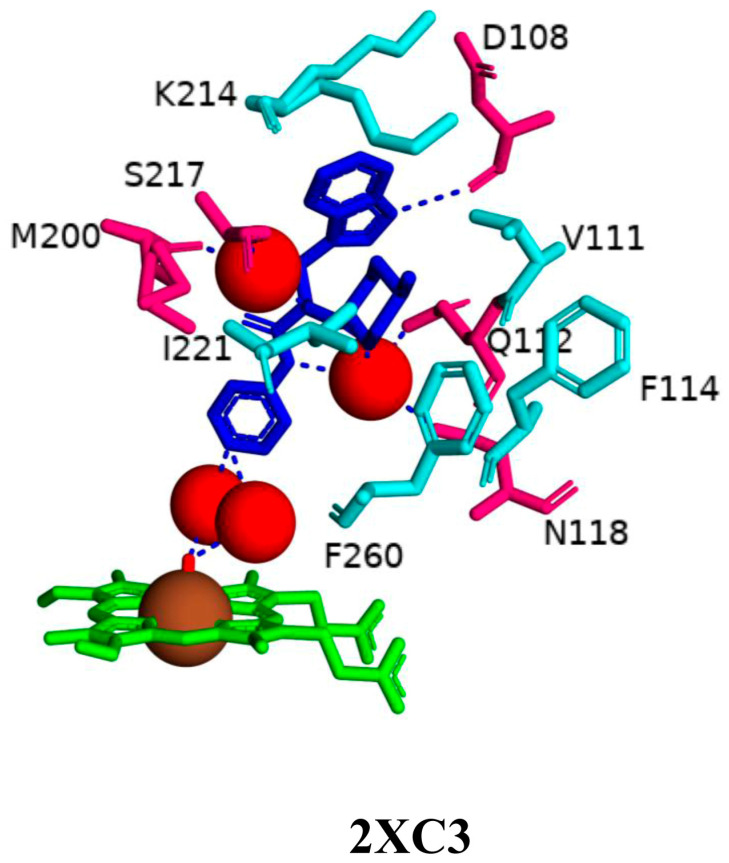
Analysis of CYP125A1 interactions with LP10 molecule (α-[(4-methylcyclohexyl)carbonyl amino]-N-4-pyridinyl-1H-indole-3-propanamide). Heme is shown in green, iron is shown as a brown sphere, and the ligand LP10 is shown in blue. Amino acid residues sharing a direct and water-mediated polar interaction with the ligand are shown in pink, and amino acid residues sharing a hydrophobic interaction with the ligand are shown in cyan. Polar interactions are indicated as blue dashed lines, water molecules are represented as red spheres, and amino acid residues are labelled according to their single-letter codes. The PDB code is indicated under the structure. Amino acids found within 5 Å of the ligand are shown in Table 5.

Analysis of CYP125A1 interactions with a non-azole compound, LP10 molecule (α-[(4-methylcyclohexyl)carbonyl amino]-N-4-pyridinyl-1H-indole-3-propanamide), further highlighted the active site cavity shape of this P450, especially the lipophilic tunnel, which plays a crucial role in determining the interactions between a ligand and heme iron [38]. The LP10 molecule completely shifted CYP125A1 to its low-spin state and has a lower affinity compared to cholesterol or cholest-4-en-3-one [38]. Binding of the LP10 molecule to CYP125A1 did not result in complete inhibition of activity, further indicating that the interactions are not strong. This was evident in the crystal structures, where LP10 binding does not affect conformational changes, as it is unable to penetrate deeply into the narrow tunnel of the active site cavity [38] (Figure 3). Binding mode revealed that the LP10 molecule inhabited the empty surrounding pocket seen in the CYP125A1-cholest-4-en-3-one complex and the gap between the B, G, and I helices that are typically occupied by the tetracyclic steroid nucleus [24,38]. Although the methylcyclohexyl and indole moieties of LP10 were oriented toward the protein surface, the pyridinyl ring was pointing toward the heme. LP10 was unable to fully penetrate the narrow tunnel that accommodates the aliphatic chain of cholest-4-en-3-one [24].

The amide nitrogen atoms of LP10 interacted with the heme iron via two water molecules (Figure 3). Several amino acids have been identified as interacting with the LP10 molecule. Phe100, Val111, Gln112, and Lys214 were in the closest contact with the indole ring within the active site cavity. The indole aromatic nitrogen atom of LP10 forms a polar contact with Asp108 [38]. Polar contact with two water molecules via its amide nitrogen atoms was also revealed, leading to water-mediated polar interactions with Gln112, Asn118, Met200, and Ser217 (Figure 3). The methyl group on the cyclohexane ring formed extensive hydrophobic interactions with the Val111, Phe114, Phe260, Ile221, Ser217, and Lys214 residues. The terminal oxygen and nitrogen atoms of Ser217 and Lys214, respectively, pointed away from the inhibitor. The methyl group was found to play a key role in binding to CYP125A1, as its removal resulted in a seven-fold decrease in binding affinity [38]. Lys214 is not involved in the formation of a salt bridge with Asp108 in the CYP125A1-LP10 complex compared to the CYP125A1-cholest-4-en-3-one complex [38]. Furthermore, the CYP125A1 active site cavity was found to be highly hydrophobic due to the presence of amino acids such as Leu117, Ala268, Val313, Phe316, and the methyl group of Thr272.

Analysis of CYP125A1-LP10 and ligand-free crystal structures in the ferric resting state revealed that binding of the LP10 molecule forces a water molecule into coordination with the heme iron, resulting in a type I inhibition and thus a low-spin state. Without LP10, the oscillation of the iron axial water molecule between the coordinated and non-coordinated positions results in CYP125A1 in a mixed low-spin/high-spin form. The interaction of the LP10 molecule with CYP125A1 contradicts the well-established phenomenon that the incoming ligand displaces the axial water. These study results indicate that any inhibitor developed against this P450 should be able to penetrate the lipophilic tunnel; thus, it should have an elongated structure that can reach deep inside the highly hydrophobic active site cavity.

### 2.4. Indole-Derived Inhibitors Interact Differently Compared to the Steroids

In a previous study, a thorough analysis of CYP125A1 interactions with derivatives of ethyl 5-(pyridin-4-yl)-1H-indole-2-carboxylate led to the identification of potential anti-TB inhibitors [34]. Additionally, it was revealed that these compounds uniquely bind to CYP125A1 compared to the substrate cholest-4-en-3-one [34]. A total of 19 compounds were synthesized (Table 6), and their structure–activity analysis against CYP125A1 and in vitro growth inhibition activity against *M. tuberculosis* H37Rv, MDR- and XDR-strains were carried out. Here, a detailed analysis of the interactions of the different compound CYP125A1 is presented.

**Figure 4:**
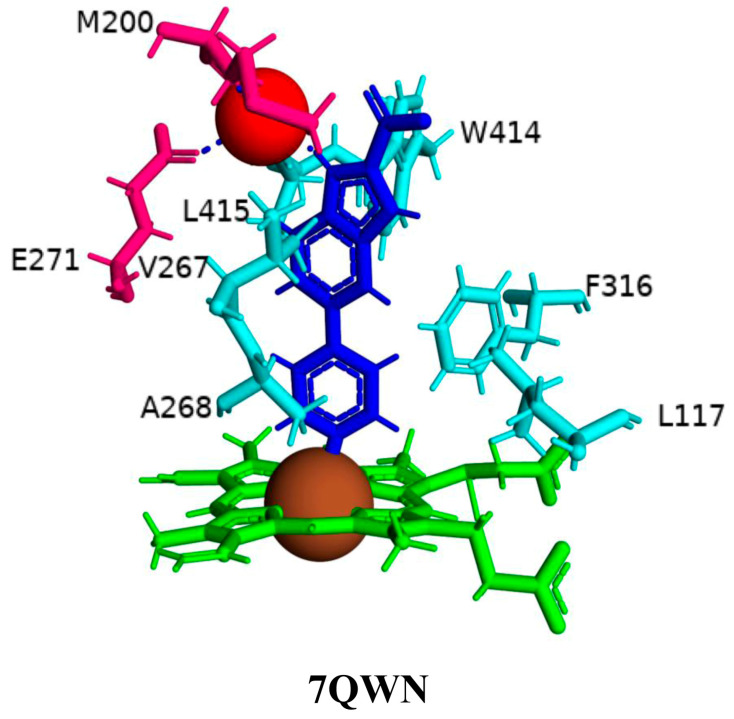
Analysis of CYP125A1 interactions with compound **4** (ethyl 5-pyridin-4-yl-1~(H)-indole-2-carboxylate). Heme is shown in green, iron is shown as a brown sphere, and the ligand is shown in blue. Amino acid residues sharing a direct and water-mediated polar interaction with the ligand are shown in pink, and amino acid residues sharing a hydrophobic interaction with the ligand are shown in cyan. Polar interactions are indicated as blue dashed lines, water molecules are represented as red spheres, and amino acid residues are labelled according to their single-letter codes. The PDB code is indicated under the structure. Amino acids found within 5 Å of the ligand are shown in Table 5.

The binding mode of compounds **1**–**4** was found to be the same, where a large number of amino acids, including Leu117, Val267, Ala268, Phe316, Trp414, and Leu415, interacted hydrophobically with these compounds (Figure 4). Compound **4** interacts with CYP125A1 such that it is perpendicular to the heme with pyridine towards the heme, and the indole occupies a pocket formed by the amino acids Val267, Glu271, Trp414, and Leu415. The binding mode of this indole derivative is unique, as this small pocket is not observed when CYP125A1 interacts with cholestenone [24]. The indole nitrogen formed solvent-mediated polar interactions where a water molecule coordinated between the carboxyl oxygen of Met200 and the side chain of Glu271 (Figure 4).

**Figure 5:**
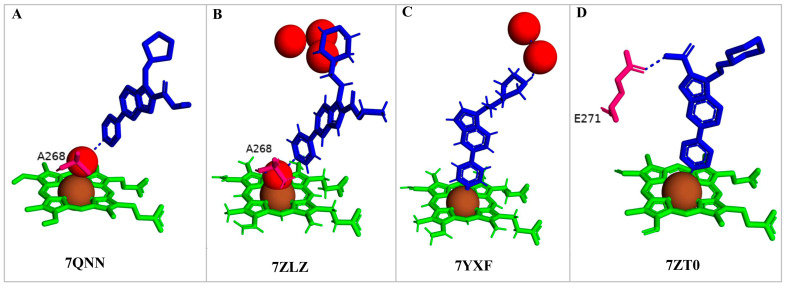
Analysis of CYP125A1 interaction with an indole inhibitor substituted with different rings in the cyclohexyl group. CYP125 in complex with an indole inhibitor containing cyclopentyl (compound **8**) (**A**), morpholine (compound **12**) (**B**), piperazine (compound **14**) (**C**), and piperazine with a functional group, carboxamide (compound **15**) (**D**). Heme is shown in green, iron is shown as a brown sphere, and the ligand is shown in blue. Amino acid residues sharing a direct and water-mediated polar interaction with the ligand are shown in pink. Polar interactions are indicated as blue dashed lines, water molecules are represented as red spheres, and amino acid residues are labeled according to their single-letter codes. The PDB code is displayed under the structure. Amino acids found within 5 Å of the ligand are shown in Table 5.

Compounds **7**, **8**, **10**, and **12** bind to CYP125A1 such that their pyridine is coordinated via a water molecule and thus produce reverse-type I interactions, similar to those observed with LP10 (as discussed above). The analysis of CYP125A1 interactions with compound **8** revealed that the nitrogen atom of the pyridine ring showed a water-mediated interaction with the heme and Ala268 (Figure 5A). The ligands that are seen to be farther away from the heme are usually coordinated by a pyridine molecule, facilitated by a water molecule. This indicated that the elaboration from the R1 position (N of the indole) affected the binding of these compounds at a greater distance from the heme. Phe100 appears to block compounds **7** and **8** from entering the active site cavity more deeply due to the presence of an aliphatic ring on the methylene linker. Compounds **10** and **12**, due to the presence of a more extended linker at the R1 position, bind closer to the heme. Three amino acids, Ile97, Phe100, and Thr201, were found to be blocking the deeper penetration of compounds **7** and **8** into the active site cavity. Furthermore, Thr201 was found to form a hydrogen bond with the side chain of Glu271 in the CYP125A1-complex 7, and this was not observed in CYP125A1-complex 4. This hydrogen bond between the two amino acids appears to further restrict the entry of compound **7**.

Compound **12** forms a direct hydrogen bond with one water molecule via the nitrogen atom of morpholine, leading to water-mediated polar interactions with two additional water molecules (Figure 5B). The nitrogen atom of the pyridine directly coordinated with heme (type II interaction) in compounds **14** and **15**, but the orientation is rotated almost 180° around the heme vertical axis relative to compound **4** (Figure 5C, D). Compound **14** formed a polar contact with the water molecule, leading to water-mediated interaction with the second water molecule (Figure 5C). Compound **15** exhibited a polar interaction with the heme iron through the nitrogen atom of the pyridine. The NH of carboxamide forms a direct interaction with Glu271 (Figure 5D). The amide group of compound **15** is found to make a hydrogen bond with the carboxyl side chain of Glu271, and this interaction increases compound **15**’s affinity compared to compound **14**, which lacks this amide group [34].

**Figure 6:**
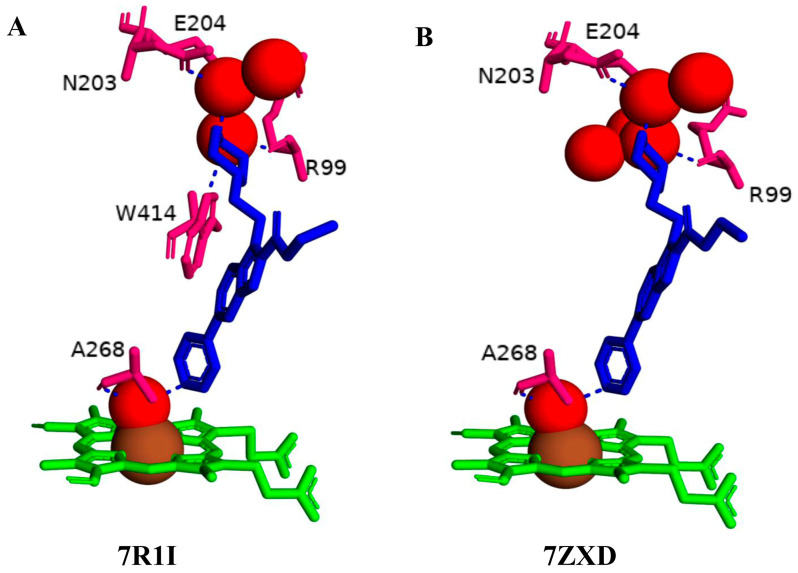
Analysis of CYP125A1 interaction with the indole inhibitor containing piperidine in both compounds and functional group carboxylate (compound **10**) (**A**) and ketone (compound **19**) (**B**). Heme is shown in green, iron is shown as a brown sphere, and the ligand is shown in blue. Amino acid residues sharing a direct and water-mediated polar interaction with the ligand are shown in pink. Polar interactions are indicated as blue dashed lines, water molecules are represented as red spheres, and amino acid residues are labeled according to their single-letter codes. The PDB code is indicated in brackets under the structure. Amino acids found within 5 Å of the ligand are shown in Table 5.

Compounds **10** and **19** interacted with CYP125A1 with the same binding mode as compounds **8** and **12**, where the nitrogen atom of the pyridine primarily coordinates the heme via the sixth water ligand and, to a lesser extent, directly to the heme iron and forms a water-mediated interaction with Ala268 (Figure 6A, B). The NH of the piperidine moiety in both structures forms water-mediated interactions with Arg99, Asn203, and Glu204. The NH of piperidine in compound **10**, containing a carboxyl group, additionally forms a water-mediated bond with Trp414 (Figure 6A). This water-mediated bond helps to stabilize the indole molecule in the active site, preventing the enzyme from binding its natural substrate. Compounds **19** and **10** have been optimized towards the observed pose of compound **8** in CYP125, with changes that improved solubility and allowed more favorable interactions with the more polar environment around Asn203, Glu204, the carboxyl oxygen of Arg99, and the NH of Trp414. It was noticed that elaboration from the N of the indole significantly alters the binding posture of these evolved series when analyzing the corresponding CYP125 ligand complex X-ray crystal structures [34].

## 3. Materials and Methods

### 3.1. Retrieving the Crystal Structures of CYP125 P450s

Twenty-one CYP125A P450 family members’ protein crystal structures are available for public use at RCSB PDB [35] and were used in this study (Table 2). Information on CYP125A P450s used in the study is provided in Table 2.

### 3.2. Assigning Crystal Structures of CYP125 into an Open and Closed Conformation

CYP125 crystal structures were analyzed and assigned individually to either an open (containing only the heme cofactor) or closed (bound with ligand and heme) conformation. The active site surface area and volume of each open and closed CYP125 crystal structure were analyzed using the Computed Atlas of Surface Topography of Proteins (CASTp) Version 3.0 [41]. Each of the individual PDBs of the CYP125 crystal structure was uploaded to the CASTp server, and after the server completed the analysis, the volume and area of the active site cavity were noted.

### 3.3. Analysis of CYP125 Active Site

To analyze the active site, each CYP125 PDB file was uploaded separately into the PyMOL software, Version 2.2.5 [42]. The heme represented the active site in open conformation, and in closed conformation, it was represented by the heme and ligand within the binding pocket. The active site cavities were identified using heme as the binding pocket’s center point, and amino acid residues falling within 5 Å were chosen as described previously [31,32,39]. The amino acid residues for both open and closed conformation were analyzed. Next, the amino acid residues for each PDB were counted and compared to identify any changes in the active site composition. Accordingly, conserved amino acids and those unique to either open or closed confirmation were identified. In the figures, the amino acid residues are represented as sticks and labeled using one-letter amino acid codes.

### 3.4. Analysis of CYP125A-Ligand Interactions

Out of the 21 CYP125A crystal structures, 15 were in closed conformation. As previously mentioned, each PDB file was uploaded to PyMOL software, Version 2.2.5 [42], and the active site cavity was chosen. For an extended bound ligand out of the selected binding pocket, all interacting amino acid residues were included by choosing 5 Å. In the figure, amino acid residues are represented as sticks and labeled with their corresponding single-letter amino acid codes. Polar contacts with any atoms were then selected. If ligand interactions with specific amino acid residues were determined, dashed lines would appear connecting the ligand and the specific amino acid residue, water molecule, or solvent molecule. If the ligand interacted with a water molecule, polar interactions with the water molecule were selected to analyze water-mediated bonds with the ligand. For a ligand that interacts with a water molecule, polar interactions between the ligand and water were identified to analyze water-mediated bonds with the ligand. Based on known data and literature, hydrophobic residues within a 5 Å radius were selected and are shown as sticks. The amino acid residues that do not interact with the ligand were removed from the figures.

### 3.5. Annotation of P450 Characteristic Secondary Structures and Identification of Substrate Recognition Sites (SRSs)

The P450 characteristics and identities for the CYP125A1 alpha helices and beta sheets were carried out as previously described [43]. The CYP125A1 PDB file (2X5L) was chosen and uploaded to PyMOL software, Version 2.2.5 [42]. The alpha helices and beta sheets were assigned in red and blue font colors, respectively, to map the secondary structural components. Alpha helices and beta sheets were subsequently named as per the P450 notations, as described elsewhere [43]. The identification of substrate recognition sites (SRSs) was carried out as described elsewhere [44]. SRS1 was mapped between alpha helices B and C along the BC-loop, SRS2 and SRS3 were found in the C-terminal end of alpha helices F and G, respectively, and SRS4 was located in the center portion of the I helix. SRS5 was found within beta sheet β_1–4_, and SRS6 was located in the beta sheet between β_4-1_ and β_4-2_.

## 4. Conclusions

**Figure 7:**
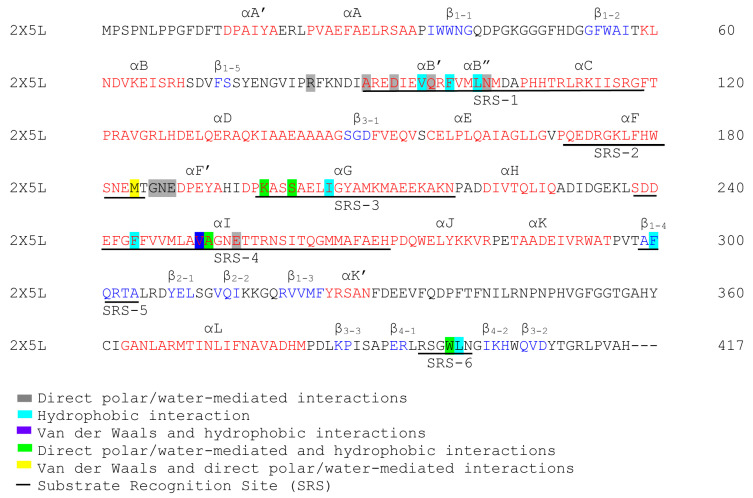
P450 characteristics’ secondary structural analysis of CYP125A members. The amino acid residues that form part of alpha helices and beta sheets are indicated as red and blue, respectively. The roles of various amino acids are illustrated with different-colored backgrounds and described in the text.

CYP125A1 of *Mycobacterium tuberculosis* is a well-known drug target, and research is underway to develop inhibitors against this P450 enzyme. Comprehensive analysis of CYP125A crystal structures revealed that the binding of the ligands in the active site cavity decreases the surface area, indicating a dynamic change in the active site cavity when there is a bound ligand. The dynamic structural changes revealed that CYP125A members are somewhat flexible compared to CYP121A1, but not as much as CYP102A1, CYP109E1, and CYP107FH5. Structural dynamic analysis between open and closed conformations of the crystal structures revealed amino acids that are involved in maintaining the structure of the active site and in catalysis, including interacting with substrates and inhibitors (Figure 7). It is noteworthy that most of the amino acids involved in interacting with ligands (substrates or inhibitors) and in catalysis can be found in six substrate recognition sites (SRSs) (Figure 7), indicating the importance of these regions in this and other P450s. The shape of the active site cavity of CYP125A1 is found to physically hinder the entry of molecules, thereby determining substrate or inhibitor specificity. Interestingly, substrates and inhibitors are found to bind in two distinct locations within the active site cavity. The mechanism employed by CYP125A1 regarding substrate and inhibitor specificity differs from that of CYP109 P450s, where substrate specificity depends on the orientation and constituents present on the substrate [40].

One of the unique findings is that some indole-derived inhibitors and the non-azole inhibitor LP10 did not displace the water molecule; rather, the water molecule was involved in the coordination between heme-iron and the inhibitor. Hydrophobic interactions with amino acids in the active site are found to stabilize the enzyme-inhibitor complex, thereby enhancing the inhibitor’s binding affinity to the enzyme. It is noted that any inhibitor developed to target this P450 should be able to enter the lipophilic tunnel; hence, it should have an elongated structure that extends deep into the extremely hydrophobic active site cavity.

The notion is that CYP125 essentiality is conditional, as the presence of compensatory enzymes such as CYP142A1 would not be an ideal condition for using CYP125 as a drug target. However, CYP125 P450s can be a standalone drug target against pathogens that lack these compensatory enzymes, or can be a combinatorial drug target along with compensatory enzymes such as CYP142A1, due to the functional redundancy. This study provides comprehensive information on the mycobacterial CYP125A1 structure–function analysis, highlighting the dynamics of the active site cavity and the role of amino acids in catalysis. The study results will assist researchers in designing new and targeted CYP125A1 inhibitors.

## Figures and Tables

**Table 1:** Functional analysis of CYP125 P450s from different bacterial species.

Ligands	Species Code and P450									
	*Mtb* H37Rv CYP125A1	*Msmeg* MC2 155 CYP125A3	*Msmeg* MC2 155 CYP125A4	*Rjos* CYP125	*Mbov* ATCC BAA-935/AF2122/97 CYP125	*Mtb* CDC1551 CYP125A1	*Save* MA4680 CYP125A2	*Speu* ATCC27952 CYP125A13	*Mmar* CYP125A6	*Mmar* CYP125A7
Cholesterol	√	√	√	√	√	√	-	√	√	√
Cholest-4-en-3-one	√	√	√	-	-	√	-	-	√	√
7α-hydroxycholesterol	√	√	√	-	-	-	-	-	√	-
7α-hydroxy-4-cholesten-3-one	√	X	√	-	-	-	-	-	-	-
Chrysin (5,7-dihydroxyisoflavone)	-	-	-	-	-	-	√	-	-	-
Cholesteryl sulfate	-	-	-	-	-	-	-	-	√	-
5β-cholestan-3α-ol	-	-	-	-	-	-	-	-	√	√
5β-cholestan-3β-ol	-	-	-	-	-	-	-	-	√	√
cholesterol-5α,6α-epoxide	-	-	-	-	-	-	-	-	√	√
7-ketocholesterol	-	-	-	-	-	-	-	-	√	√
7β-hydroxycholesterol	-	-	-	-	-	-	-	-	√	√
7-dehydrocholesterol	-	-	-	-	-	-	-	-	√	-
Sitosterol	-	-	-	-	-	-	-	-	√	
5α-cholestan-3β-ol	-	-	-	-	-	-	-	-	-	√
Cholesterol-5α,6α-epoxide	-	-	-	-	-	-	-	-	-	-
References	[20,23,24,25]	[25,26]	[25,26]	[21]	[22]	[24]	[27]	[28]	[29,30]	[29,30]

Abbreviations: *Mtb* H37Rv: *Mycobacterium tuberculosis* H37Rv; *Msmeg* MC2 155: *Mycobacterium smegmatis* MC2 155; *Rjos*: *Rhodococcus jostii* strain RHA1; *Mbov* ATCC BAA-935/AF2122/97: *Mycobacterium bovis* strain ATCC BAA-935/AF2122/97; *Mtb* CDC1551: *Mycobacterium tuberculosis CDC1551; Save* MA4680: *Streptomyces avermitilis* MA4680; *Speu* ATCC27952: *Streptomyces peucetius* ATCC27952; *Mmar*: *Mycobacterium marinum*; Symbols: √, Hydroxylation activity; X, No Activity; -, Not tested.

**Table 4:** Amino acid dynamics analysis for CYP125A P450s. Amino acid residues within 5 Å of the heme were selected. Amino acids that are conserved, unique, and part of the active site were presented for both the open and closed conformations. The amino acid count is given in parentheses.

P450	Confirmation	PDB Code	Number of Amino Acids in the Active Site Cavity	Common Amino Acids Among Open/Closed Conformations	Unique Amino Acids Among Open/Closed Conformations	Common Amino Acids Between Open and Closed Conformations	Unique Amino Acids in Open or Closed Confirmation
CYP125A1	Open	2X5L	34	Ser83, Met116, Leu117, His124, Arg128, Phe135, Ile179, Leu182, Met264, Leu265, Ala268, Gly269, Thr272, Thr273, Ser276, Val307, Pro312, Val313, Phe316, Arg318, Tyr341, Gly368, Phe369, Gly370, Gly371, Ala374, His375, Tyr376, Cys377, Ile378, Gly379, Leu382, Ala383, Ile387 (34)	-	Ser83, Met116, Leu117, His124, Arg128, Phe135, Ile179, Leu182, Met264, Leu265, Ala268, Gly269, Thr272, Thr273, Ser276, Val307, Pro312, Val313, Phe316, Arg318, Tyr341, Gly368, Phe369, Gly370, Gly371, Ala374, His375, Tyr376, Cys377, Ile378, Gly379, Leu382, Ala383, Ile387 (34)	
	Open	2XN8	34		-		
	Open	3IVY	34		-		
	Open	3IW0	34		-		
	Closed	2X5W	34	Met116, Leu117, His124, Arg128, Phe135, Ile179, Met264, Leu265, Ala268, Gly269, Thr272, Thr273, Ser276, Val307, Pro312, Val313, Phe316, Arg318, Tyr341, Gly368, Phe369, Gly370, Ala374, His375, Tyr376, Cys377, Ile378, Gly379, Leu382, Ala383, Ile387 (31)	Ser83, Gly371, Thr372 (3)		Ser91, Val96, Ile131, Pro175, Met339, Ala380, Ala178, Phe261, Asn270, Thr311, Thr372, Val367 (12)
	Closed	2XC3	34		Ser83, Leu182, Gly371 (3)		
	Closed	3IW1	35		Ser83, Leu182, Val367, Gly371 (4)		
	Closed	3IW2	37		Ser91, Val96, Pro175, Met339, Val367, Ala380 (6)		
	Closed	7QKE	45		Ser83, Ser91, Val96, Ile131, Pro175, Ala178, Leu182, Phe261, Asn270, Thr311, Met339, Val367, Gly371, Ala380 (14)		
	Closed	7QNN	34		Ser83, Leu182, Gly371 (3)		
	Closed	7QWN	42		Ser83, Ile131, Pro175, Leu182, Phe261, Asn270, Thr311, Met339, Val367, Gly371, Ala380 (11)		
	Closed	7R1I	36		Ser83, Leu182, Phe261, Met339, Gly371 (5)		
	Closed	7R3U	44		Ser83, Val96, Ile131, Pro175, Ala178, Leu182, Phe261, Asn270, Thr311, Met339, Val367, Gly371, Ala380 (13)		
	Closed	7YXF	42		Ser83, Val96, Ile131, Pro175, Leu182, Asn270, Thr311, Met339, Val367, Gly371, Ala380 (11)		
	Closed	7ZLZ	44		Ser83, Val96, Ile131, Pro175, Ala178, Leu182, Phe261, Asn270, Thr311, Met339, Val367, Gly371, Ala380 (13)		
	Closed	7ZQR	36		Ser91, Val96, Leu182, Met339, Val367 (5)		
	Closed	7ZSU	42		Ser83, Ser91, Val96, Ile131, Leu182, Phe261, Thr311, Met339, val367, Gly371, Ala380 (11)		
	Closed	7ZT0	34		Ser83, Ile182, Gly371 (3)		
	Closed	7ZXD	35		Ser83, Leu182, Val367, Gly371 (4)		

**Table 6:** Information on indole-derived inhibitors, their assigned number, and the CYP125A1 crystal structure (PDB code) that interacts with the specific inhibitor.

PDB Code	Compound Number	Compound Name	Compound Structure
7R3U	**1**	1-[4-(1,2,3-thiadiazol-4-yl)phenyl]methanamine	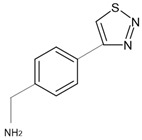
7ZQR	**2**	4-(4-methoxyphenyl)pyridine	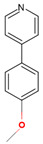
7ZSU	**3**	methyl 3-pyridin-4-ylbenzoate	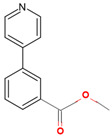
7QWN	**4**	ethyl 5-pyridin-4-yl-1~(H)-indole-2-carboxylate	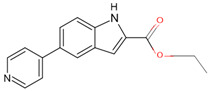
7QKE	**7**	ethyl 1-(cyclohexylmethyl)-5-pyridin-4-yl-indole-2-carboxylate	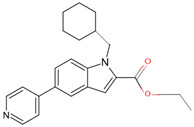
7QNN	**8**	ethyl 1-(cyclopentylmethyl)-5-pyridin-4-yl-indole-2-carboxylate	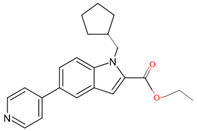
7R1I	**10**	ethyl 1-(2-piperidin-4-ylethyl)-5-pyridin-4-yl-indole-2-carboxylate	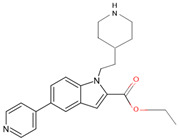
7ZLZ	**12**	ethyl 1-(2-morpholin-4-ylethyl)-5-pyridin-4-yl-indole-2-carboxylate	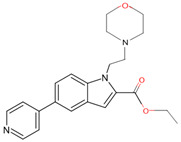
7YXF	**14**	1-(2-piperazin-1-ylethyl)-5-pyridin-4-yl-indole	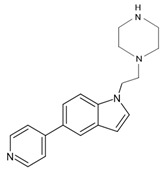
7ZT0	**15**	1-(2-piperazin-1-ylethyl)-5-pyridin-4-yl-indole-2-carboxamide	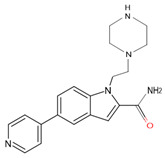
7ZXD	**19**	1-[1-(2-piperidin-4-ylethyl)-5-pyridin-4-yl-indol-2-yl]butan-1-one	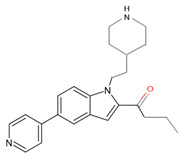

## Data Availability

Data are contained within the article.

## References

[1] Zhang S.X., Miao F.Y., Yang J., Zhou W.T., Lv S., Wei F.N., Wang Y., Hu X.J., Yin P., Zheng P.Y. (2024). Global, regional, and national burden of HIV-negative tuberculosis, 1990–2021: Findings from the Global Burden of Disease Study 2021. Infect. Dis. Poverty.

[2] WHO (2024). Global Tuberculosis Report 2024.

[3] Olivier C., Luies L. (2023). WHO Goals and Beyond: Managing HIV/TB Co-infection in South Africa. SN Compr. Clin. Med..

[4] Perveen S., Kumari D., Singh K., Sharma R. (2022). Tuberculosis drug discovery: Progression and future interventions in the wake of emerging resistance. Eur. J. Med. Chem..

[5] Bernhardt R. (2006). Cytochromes P450 as versatile biocatalysts. J. Biotechnol..

[6] Isin E.M., Guengerich F.P. (2007). Complex reactions catalyzed by cytochrome P450 enzymes. Biochim. Biophys. Acta.

[7] Guengerich F.P., Munro A.W. (2013). Unusual cytochrome p450 enzymes and reactions. J. Biol. Chem..

[8] Urlacher V.B., Girhard M. (2019). Cytochrome P450 Monooxygenases in Biotechnology and Synthetic Biology. Trends Biotechnol..

[9] Ngcobo P.E., Nkosi B.V.Z., Chen W., Nelson D.R., Syed K. (2023). Evolution of cytochrome P450 enzymes and their redox partners in Archaea. Int. J. Mol. Sci..

[10] Gront D., Syed K., Nelson D.R. (2025). Exploring P450 superfamily diversity with P450Atlas-Online tool for automated subfamily assignment. Protein Sci..

[11] Zondo N.M., Padayachee T., Nelson D.R., Syed K. (2023). Saprophytic to pathogenic mycobacteria: Loss of cytochrome P450s vis a vis their prominent involvement in natural metabolite biosynthesis. Int. J. Mol. Sci..

[12] van Wyk R., van Wyk M., Mashele S.S., Nelson D.R., Syed K. (2019). Comprehensive comparative analysis of cholesterol catabolic genes/proteins in mycobacterial species. Int. J. Mol. Sci..

[13] Parvez M., Qhanya L.B., Mthakathi N.T., Kgosiemang I.K., Bamal H.D., Pagadala N.S., Xie T., Yang H., Chen H., Theron C.W. (2016). Molecular evolutionary dynamics of cytochrome P450 monooxygenases across kingdoms: Special focus on mycobacterial P450s. Sci. Rep..

[14] Cole S.T., Brosch R., Parkhill J., Garnier T., Churcher C., Harris D., Gordon S.V., Eiglmeier K., Gas S., Barry C.E. (1998). Deciphering the biology of *Mycobacterium tuberculosis* from the complete genome sequence. Nature.

[15] Ortiz de Montellano P.R. (2018). Potential drug targets in the *Mycobacterium tuberculosis* cytochrome P450 system. J. Inorg. Biochem..

[16] Chang J.C., Harik N.S., Liao R.P., Sherman D.R. (2007). Identification of Mycobacterial genes that alter growth and pathology in macrophages and in mice. J. Infect. Dis..

[17] Chang J.C., Miner M.D., Pandey A.K., Gill W.P., Harik N.S., Sassetti C.M., Sherman D.R. (2009). igr Genes and Mycobacterium tuberculosis cholesterol metabolism. J. Bacteriol..

[18] Frank D.J., Zhao Y., Wong S.H., Basudhar D., De Voss J.J., Ortiz de Montellano P.R. (2016). Cholesterol Analogs with Degradation-resistant Alkyl Side Chains Are Effective Mycobacterium tuberculosis Growth Inhibitors. J. Biol. Chem..

[19] Griffin J.E., Gawronski J.D., Dejesus M.A., Ioerger T.R., Akerley B.J., Sassetti C.M. (2011). High-resolution phenotypic profiling defines genes essential for mycobacterial growth and cholesterol catabolism. PLoS Pathog..

[20] McLean K.J., Lafite P., Levy C., Cheesman M.R., Mast N., Pikuleva I.A., Leys D., Munro A.W. (2009). The Structure of Mycobacterium tuberculosis CYP125: Molecular basis for cholesterol binding in a P450 needed for host infection. J. Biol. Chem..

[21] Rosłoniec K.Z., Wilbrink M.H., Capyk J.K., Mohn W.W., Ostendorf M., van der Geize R., Dijkhuizen L., Eltis L.D. (2009). Cytochrome P450 125 (CYP125) catalyses C26-hydroxylation to initiate sterol side-chain degradation in Rhodococcus jostii RHA1. Mol. Microbiol..

[22] Capyk J.K., Kalscheuer R., Stewart G.R., Liu J., Kwon H., Zhao R., Okamoto S., Jacobs W.R., Eltis L.D., Mohn W.W. (2009). Mycobacterial cytochrome p450 125 (cyp125) catalyzes the terminal hydroxylation of c27 steroids. J. Biol. Chem..

[23] Johnston J.B., Ouellet H., Ortiz de Montellano P.R. (2010). Functional redundancy of steroid C26-monooxygenase activity in Mycobacterium tuberculosis revealed by biochemical and genetic analyses. J. Biol. Chem..

[24] Ouellet H., Guan S., Johnston J.B., Chow E.D., Kells P.M., Burlingame A.L., Cox J.S., Podust L.M., de Montellano P.R. (2010). *Mycobacterium tuberculosis* CYP125A1, a steroid C27 monooxygenase that detoxifies intracellularly generated cholest-4-en-3-one. Mol. Microbiol..

[25] Frank D.J., Waddling C.A., La M., Ortiz de Montellano P.R. (2015). Cytochrome P450 125A4, the Third Cholesterol C-26 Hydroxylase from *Mycobacterium smegmatis*. Biochemistry.

[26] García-Fernández E., Frank D.J., Galán B., Kells P.M., Podust L.M., García J.L., Ortiz de Montellano P.R. (2013). A highly conserved mycobacterial cholesterol catabolic pathway. Environ. Microbiol..

[27] Pandey B.P., Lee N., Choi K.Y., Jung E., Jeong D.H., Kim B.G. (2011). Screening of bacterial cytochrome P450s responsible for regiospecific hydroxylation of (iso)flavonoids. Enzym. Microb. Technol..

[28] Rimal H., Subedi P., Kim K.H., Park H., Lee J.H., Oh T.-J. (2020). Characterization of CYP125A13, the First Steroid C-27 Monooxygenase from Streptomyces peucetius ATCC27952. J. Microbiol. Biotechnol..

[29] Doherty D.Z., Ghith A., Ho A., De Voss J.J., Bell S.G. (2023). The bacterial cytochrome P450 (CYP) CYP125 enzymes can competitively oxidise sitosterol in the presence of cholesterol. Chem. Commun..

[30] Ghith A., Bruning J.B., Bell S.G. (2023). The oxidation of cholesterol derivatives by the CYP124 and CYP142 enzymes from *Mycobacterium marinum*. J. Steroid Biochem. Mol. Biol..

[31] Padayachee T., Lamb D., Nelson D., Syed K. (2024). Structure–Function Analysis of the Essential Mycobacterium tuberculosis P450 Drug Target, CYP121A1. Int. J. Mol. Sci..

[32] Padayachee T., Lamb D.C., Nelson D.R., Syed K. (2023). Structure-Function Analysis of the Biotechnologically Important Cytochrome P450 107 (CYP107) Enzyme Family. Biomolecules.

[33] Wankhade G., Kamble S., Deshmukh S., Jena L., Waghmare P., Harinath B. (2017). Inhibition of mycobacterial CYP125 enzyme by sesamin and [beta]-sitosterol: An in silico and in vitro study. Biomed. Biotechnol. Res. J..

[34] Katariya M., Snee M., Tunnicliffe R., Kavanagh M., Boshoff H., Amadi Obasi C., Levy C., Munro A., Abell C., Leys D. (2023). Structure Based Discovery of Inhibitors of CYP125 and CYP142 from *Mycobacterium tuberculosis*. Chem. Weinh. Der Bergstr. Ger..

[35] Berman H.M., Westbrook J., Feng Z., Gilliland G., Bhat T.N., Weissig H., Shindyalov I.N., Bourne P.E. (2000). The Protein Data Bank. Nucleic Acids Res..

[36] Ahmed S., Smith J., Nicholls P., Whomsley R., Cariuk P. (1995). Synthesis and biological evaluation of imidazole based compounds as cytochrome P-450 inhibitors. Drug Des. Discov..

[37] Urban P., Lautier T., Pompon D., Truan G. (2018). Ligand Access Channels in Cytochrome P450 Enzymes: A Review. Int. J. Mol. Sci..

[38] Ouellet H., Kells P.M., Ortiz de Montellano P.R., Podust L.M. (2011). Reverse type I inhibitor of *Mycobacterium tuberculosis* CYP125A1. Bioorg Med. Chem. Lett..

[39] Padayachee T., Lamb D.C., Nelson D.R., Syed K. (2025). Structure-Function Analysis of the Self-Sufficient CYP102 Family Provides New Insights into Their Biochemistry. Int. J. Mol. Sci..

[40] Msweli S.M., Padayachee T., Khumalo T., Nelson D.R., Lamb D.C., Syed K. (2025). Structure–Function Analysis of the Steroid-Hydroxylating Cytochrome P450 109 (CYP109) Enzyme Family. Int. J. Mol. Sci..

[41] Binkowski T.A., Naghibzadeh S., Liang J. (2003). CASTp: Computed Atlas of Surface Topography of proteins. Nucleic Acids Res..

[42] Schrödinger L.D. (2020). The PyMOL Molecular Graphics System, Version 2.0.

[43] Graham S.E., Peterson J.A. (1999). How similar are P450s and what can their differences teach us?. Arch. Biochem. Biophys..

[44] Gotoh O. (1992). Substrate recognition sites in cytochrome P450 family 2 (CYP2) proteins inferred from comparative analyses of amino acid and coding nucleotide sequences. J. Biol. Chem..

